# Behavioral effects of rhythm, carrier frequency and temporal cueing on the perception of sound sequences

**DOI:** 10.1371/journal.pone.0234251

**Published:** 2020-06-05

**Authors:** Miriam Heynckes, Peter De Weerd, Giancarlo Valente, Elia Formisano, Federico De Martino

**Affiliations:** 1 Department of Cognitive Neuroscience, Faculty of Psychology and Neuroscience, Maastricht University, Maastricht, The Netherlands; 2 Maastricht Centre for Systems Biology, Maastricht University, Maastricht, The Netherlands; Max Planck Institute for Human Cognitive and Brain Sciences, GERMANY

## Abstract

Regularity of acoustic rhythms allows predicting a target embedded within a stream thereby improving detection performance and reaction times in spectral detection tasks. In two experiments we examine whether temporal regularity enhances perceptual sensitivity and reduces reaction times using a temporal shift detection task. Participants detected temporal shifts embedded at different positions within a sequence of quintet–sounds. Narrowband quintets were centered around carrier frequencies of 200 Hz, 1100 Hz, or 3100 Hz and presented at presentation rates between 1–8 Hz. We compared rhythmic sequences to control conditions where periodicity was reduced or absent and tested whether perceptual benefits depend on the presentation rate, the spectral content of the sounds, and task difficulty. We found that (1) the slowest rate (1 Hz) led to the largest behavioral effect on sensitivity. (2) This sensitivity improvement is carrier-dependent, such that the largest improvement is observed for low-frequency (200 Hz) carriers compared to 1100 Hz and 3100 Hz carriers. (3) Moreover, we show that the predictive value of a temporal cue and that of a temporal rhythm similarly affect perceptual sensitivity. That is, both the cue and the rhythm induce confident temporal expectancies in contrast to an aperiodic rhythm, and thereby allow to effectively prepare and allocate attentional resources in time. (4) Lastly, periodic stimulation reduces reaction times compared to aperiodic stimulation, both at perceptual threshold as well as above threshold. Similarly, a temporal cue allowed participants to optimally prepare and thereby respond fastest. Overall, our results are consistent with the hypothesis that periodicity leads to optimized predictions and processing of forthcoming input and thus to behavioral benefits. Predictable temporally cued sounds provide a similar perceptual benefit to periodic rhythms, despite an additional uncertainty of target position within periodic sequences. Several neural mechanisms may underlie our findings, including the entrainment of oscillatory activity of neural populations.

## Introduction

Natural sounds are often characterized by a rhythm that enables the listener to predict and prepare for relevant events [1], a finding confirmed by many studies probing the effects of periodicity and predictability on sound perception [2–7]. Recent results have shown that periodicity and predictability of rhythms have dissociable effects on reaction time and sensitivity. Periodic (isochronous) stimuli decrease the reaction time [3,8,9], even when their rhythm is not predictive of a target [10,11]. On the other hand, predictability can increase perceptual sensitivity [5,7,12,13] even when stimulus presentation is predictable but aperiodic [6,14].

Most previous studies have focused on broadband or a few selected frequencies and have often tested presentation rates around 1.5 Hz [4,8,15–19]. This last choice is at odds with studies suggesting that the brain preferentially differentiates acoustic information covering a range of timescales through theta- (and gamma) frequency-band information [20], a result that highlights the relevance of the temporal modulations inherent in speech occurring at the syllabic-level [21,22]. The importance of testing multiple carrier frequencies and presentation rates is stressed by psychoacoustic studies on amplitude modulation suggesting that rate and carrier frequency interact [23]. To account for the interdependency between spectral and temporal processing, it has been suggested that the modulation filters modelling the auditory system [24] systematically change along the frequency axis. In particular, the auditory system may be optimized to track rapid modulations at high frequencies and slower modulations at lower carrier frequencies [25].

Electrophysiological recordings in macaque auditory cortex suggest that the mechanism engaged by rhythmic sound processing is tonotopic and thus cannot be investigated when using broadband noise bursts [26], but requires the use of narrow-band stimuli or tones [16,27]. These studies propose that the optimization of behavioral performance for periodic stimuli is achieved through the entrainment of neuronal oscillations, which temporally modulate the excitability of task-relevant neuronal populations [8,18,28,29]. Rhythmic external stimuli can entrain oscillations, during which neural delta [8] and theta oscillations [30] become aligned to the externally imposed rhythm. The entrained oscillations may form the basis of temporal predictions that can be beneficial for stimuli presented at the entrainment rhythm [19,31].

Predictions can also be formed without periodicity, by using temporally cued associations. The behavioral benefits of temporal cueing have been shown in studies on foreperiod effects [32,33]. At the behavioral level, a predictable rhythmic sequence should lead to better perceptual discrimination at predicted moments in time than a predictable single interval. This has been tested in foreperiod-paradigms where the duration of an interval is judged, either in isolated pairs or with a preceding rhythm, and results indicate that discrimination thresholds improve [13]. Studies focusing on reaction times have compared effects of temporal cueing and periodic stimulus presentation both when the predictive information was provided symbolically, showing a benefit of periodicity over symbolic cueing [34], and temporally cued not showing a difference [15,35] and suggest a cumulative benefit of combining a temporal cue and a periodic rhythm [3]. Similarly, studies using pitch discrimination tasks [36,37] have shown effects on accuracy and reaction times. Moreover, a benefit in accuracy derived from implicit predictability in temporally cued intervals has been shown [14]. In line with a recent study suggesting that delta-phase entrainment may relate differently to spectral-based and temporal sensitivity [12], it would be of interest to compare perceptual sensitivity and reaction times due to predictability derived from a periodic sequence and from a single acoustic temporal cue when using a temporal task. This would allow evaluating if a single mechanism underlies predictive effects induced by both a temporal cue and a periodic sequence.

In two behavioral studies, we systematically address these open points by investigating how rhythm and carrier frequency of periodically presented sounds contribute to the behavioral benefits of rhythmic sound perception (Experiment 1). In a pilot we compare a subset of these conditions to aperiodic sound sequences (see S1 File). In experiment 2 we compare periodic and aperiodic sound sequences and additionally test whether the predictive advantage afforded by periodicity exceeds the advantage afforded by a temporal cue (Experiment 2). Based on previous findings [6,7,12] we expect a perceptual benefit in sensitivity and reaction times of periodic over aperiodic rhythms. In addition, we hypothesize that a periodic rhythm should result in a benefit over a single temporal cue, especially when targets are presented close to perceptual threshold. With regard to the difference between various periodic rhythms, we expect behavioral improvement to occur around the peaks of the external rhythm, as opposed to a general improvement due to periodic stimulation, in line with [16]. In particular, we hypothesize that an external rhythm in the theta frequency range (~ 4 Hz), would result in improved behavioral performance compared to other presentation rates.

## Materials & methods

### Participants

Prior to testing, participants were screened for normal hearing (≤ 20 dB) at audiometric test frequencies ranging from 0.25–8 kHz. In experiment 1a and 1b, two sets of 20 participants each took part in the experiments. Two participants were excluded, one due to failure to comprehend the task and one due to discomfort caused by the loudness of the stimuli, resulting in the abortion of the experiment. As a result, we analyzed data collected from n = 19 participants for experiment 1a (15 females, 4 males) and 1b (12 females, 7 males) each, and n = 20 participants for experiment 2 (13 females, 7 males). The size of n was determined based on studies reporting similar effect sizes [6]. The Ethics Review Committee of the Faculty of Psychology and Neuroscience (ERCPN) at Maastricht University granted approval for all studies and all participants gave informed consent.

### Stimuli & design

**Fig 1:**
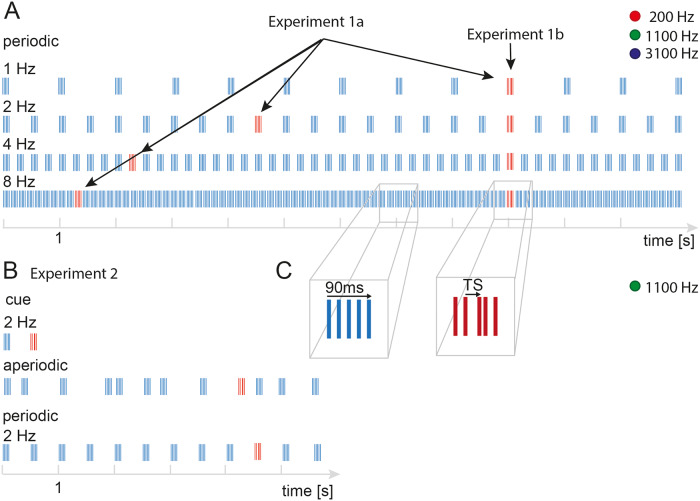
Stimuli. **A** Experiment 1. Narrowband quintets were presented at 4 different presentation rates (1,2,4,8 Hz) and 3 different carrier frequencies (200, 1100, 3100 Hz). Target position (exemplars in red) was varied between experiment 1a and 1b. In experiment 1a targets occurred in either the 9th, 10th or 11th quintet within a sequence, while in experiment 1b targets appeared at the same time across rhythms (at 9,10 or 11 s). **B** Stimuli in experiment 2 were 6s aperiodic and periodic sequences at 2 Hz, correspondingly the cue condition had an ISI of 500ms. The carrier frequency was 1100 Hz. **C**. Quintet structure. Narrowband sounds of 10ms length centered around the respective carrier frequencies were organized in a hierarchical rhythmic structure. Five sounds repeating at 50 Hz (10ms ISI) create a quintet (inset 1), while these quintets are repeated at a slow (a)periodic rhythm. Target stimuli (TS, see inset 2) had a different temporal structure: the third tone in a quintet was temporally shifted at 6ms in experiment 1a, 1b and 2 and between 1.5-7ms in experiment 3, as determined by the participant’s 70% detection threshold.

All stimulus presentation scripts were written in Matlab (The MATHWORKS Inc., Natick, MA, 234 USA), using the Psychophysics toolbox [38]. Sounds were created at a 44.1 kHz sampling rate, 16-bit resolution and delivered through Sennheiser HD650 headphones. Data and analysis scripts are publicly available (https://doi.org/10.5281/zenodo.3695583). Exemplary stimuli can be found here (https://doi.org/10.5281/zenodo.3549376). Participants were seated in a sound-attenuated chamber. Instructions were presented on a computer monitor and responses collected with a standard keyboard. Participants were asked to detect a target; a temporal shift (TS) of a narrow-band sound embedded in a sequence of quintets. Narrowband sounds were centered around carrier frequencies of 200 Hz, 1100 Hz, or 3100 Hz. The passbands around the carriers were constructed using equivalent rectangular bandwidths (ERBS = 4; [39]). Each passband consisted of a summation of 21 sinusoids with amplitude normalized to 1 and a random onset phase. A quintet consisted of five 10ms narrowband sounds, each separated by 10ms (see inset 1 Fig 1D). Targets were constructed by shifting in time the third sound in a quintet (see inset 2 Fig 1D). Depending on the experiment, this shift was either fixed at 6ms (Experiment 1) or ranged between 1.5-7ms (Experiment 2; see Table 1). In both experiments, during a trial and up to 1 second after a quintet sequence finished, participants could press a button upon detecting a TS or another button at the end of a sequence indicating they did not perceive a TS. Quintet sequences had either a periodic or aperiodic repetition of quintets or occurred in the form of a temporal cue, where a single quintet cued the following quintet that possibly contained a TS. Table 1 reports the design parameters for each experiment. The distribution of TS was non-uniform such that TS occurred in 75% of the trials. Targets appeared at one of 3 possible quintet positions within the periodic or aperiodic sequences, or in the cued quintet. In experiment 1a targets TS were presented in quintet 9, 10 or 11 (e.g. occurring after 9, 10 or 11s for the 1 Hz rhythm). With increasing rhythm, targets thereby occurred earlier in time within a sequence (the 9th quintet at 2 Hz occurs at 4.5 s). Experiment 1b controls for the earlier occurrence of targets with increasing rhythm, by keeping the time until a TS constant across rhythm (a TS occurring in the 9th quintet at 1 Hz would occur in the 18th quintet at 2 Hz). Similar to experiment 1b, TS occurred at a fixed time in both the pilot experiment preceding experiment 2 (see S1 File), as well as in experiment 2 itself.

**Table 1:** Design and overview of stimuli conditions.

	Temporal structure	rhythm	Trials/Blocks	Carrier	Task difficulty	Motivation
**Exp 1a**	Periodic	1,2,4,8 Hz	48 trials (12 per rhythm) *10 blocks	200 Hz 1100 Hz 3100 Hz	6 ms TS	Test preferred rhythm and carrier
**Exp 1b**	Periodic	1,2,4,8 Hz	48 trials (12 per rhythm) *10 blocks	200 Hz 1100 Hz 3100 Hz	6 ms TS	Rhythm effect in Exp 1a due to target position?
**Exp 2**	Periodic Aperiodic Cue	2 Hz	72 trials (24 per condition) * 6 blocks	1100 Hz	1.5–7 ms TS (70% threshold)	Periodic benefit when more difficult task?

In experiment 1a (n = 19) & 1b (n = 19) we investigated the effect of periodic rhythms (1, 2, 4, 8 Hz) and the carrier frequency (200, 1100 and 3100Hz) on target detection sensitivity (measured in d’) and reaction time. Every trial consisted of 12s periodic sequences of quintets. We conducted two versions of this experiment, in which we varied the position of the targets within the sequences. In experiment 1a we kept the number of preceding quintets until the TS constant across rhythms. This systematically reduced the time until a target appeared with increasing rhythm frequency. In experiment 1b, we kept the time at which targets appeared constant, thereby presenting an increasing number of preceding quintets with increasing rhythms prior to presenting a target quintet. The rationale to assess and compare these two experiment versions is that the strength of entrainment might increase with additional repetitions, and the effect of rhythm may be confounded by the systematic effect of target position in a sequence. Trials were counter-balanced and randomized with respect to target position and carrier frequency, and presented in (counterbalanced) blocks per rhythm, consisting of 12 trials. After four blocks (one per rhythm) subjects had a break. In total participants received 10*4 blocks, summing to a total of 480 trials. Subjects underwent a brief training session (8–12 trials) using a subset of the stimuli. During training, visual feedback on the performance was provided. Prior to the training and main experiment, participants adjusted the intensity of the sounds to equalize their perceived loudness. When comparing the three carrier frequencies at equal intensities, 200 Hz was generally perceived as softer and 3100 Hz perceived as louder relative to the reference frequency of 1100 Hz and were adjusted accordingly. These observations are in line with equal loudness contours [39].

Unless explicitly stated, the stimuli in the pilot experiment preceding experiment 2 (see S1 File) and stimuli in experiment 2 were identical to those used in experiment 1b. We decided to limit the number of conditions and chose the carrier frequency and rhythm based on our findings in experiment 1 showing that with a 2 Hz rhythm and a 1100 Hz carrier frequency behavioral performance was intermediate, and thus we could expect the behavior to be modulated by the manipulations in experiment 2. In the pilot (n = 20; 11 females, 9 males) we examined the effect of (average) rhythm on target detection, by comparing periodic predictable and aperiodic unpredictable sequences of quintets. The TS target remained fixed at 6ms. To create an aperiodic sequence, the main constraint was to present the same number of quintets in the same amount of time (compared to the periodic conditions) at aperiodic inter-stimulus intervals (ISIs). For instance, the aperiodic sequence corresponding to the 1 Hz rhythm had to be comprised of 12 quintets in 12 seconds. As a result, ISIs had to be sampled within two intervals, shorter or longer compared to the corresponding periodic condition. Periodic sequences at 1Hz have a 1s ISI, the corresponding aperiodic condition sampled ISI from two distributions with mean equal to 250ms and 1500ms. Similarly, periodic sequences at 2 Hz have a 500ms ISI, the corresponding aperiodic condition sampled ISI from two distributions with mean equal to 100ms and 733ms. In each aperiodic condition, the average over both sampled distributions approximates the periodic condition (1.07 Hz and 2.2 Hz respectively). Due to the nested temporal structure of the stimuli it was not possible to create aperiodic sequences at average rhythms of 4 Hz and 8 Hz, as the interval between quintets was too short. The participants performed 80 trials of the target detection task on aperiodic stimuli. Stimuli were presented in blocks of 16 trials, in which the average rhythm was constant.

D’ scores of the pilot experiment approached ceiling for many of the participants. Therefore, in experiment 2 (n = 20; 13 females, 7 males) we adjusted the difficulty range of the task to capture a modulation of behavior by periodicity. In addition, the stimuli were shortened to 6s, the (average) rhythm was fixed at 2 Hz and the carrier frequency at 1100 Hz, to limit the number of conditions and experiment duration. Aperiodic stimuli in experiment 2 were created similar to aperiodic stimuli in the pilot. Rhythmic stimuli (periodic predictable, aperiodic unpredictable) were compared to a temporal cue condition to test whether the predictive benefits derived from periodicity are larger than those derived from a single temporally predictive cue. Trials were presented in blocks (grouped by condition periodic, aperiodic, cue); within each block, trials were randomized, and the block order was counterbalanced across participants. Each participant completed a total of 432 trials. Task difficulty was determined through a staircase procedure preceding every block of trials, in which TS were set to achieve a behavioral performance at 70% detection threshold, as determined by means of a 2 down 1 up procedure. The termination criterion was after 200 trials or 15 reversals. The TS varied on a fixed step-size of 10 logarithmically spaced steps between 7ms and 1.5 ms (see S3 Fig for average TS size per staircase preceding a block).

### Statistical analysis

All analyses were conducted in MATLAB 2017a (The MATHWORKS Inc., Natick, MA, 234 USA). For each participant, the d’ sensitivity index of signal detection theory and mean log-reaction times (logRT) of correct trials were calculated. Reaction time was calculated relative to target onset.

Statistical analysis of reaction times and sensitivity were carried out using a Generalized Linear Mixed Model (GLMM) with Matlab’s *fitGLME* function. We assessed the model fits using likelihood ratio tests (using the function *compare* for GLMM). Contrasts were carried out performing an F-test on the specified fixed effects of the GLMM (using the function *coefTest*). Reaction times were fitted using the default identity link function, unlike the fitting of the sensitivity data for which a probit link function was used as described in more detail below.

Traditionally, d’ is estimated by counting the frequency of an observer reporting ‘yes’ conditional on the presence and absence of a signal (i.e. the hit and false alarm rates) and taking the difference of these values on a z-transformed scale [40]. In the present work, statistical group analyses on d’ are carried out using a GLMM [41] We estimate both model parameters and d’ simultaneously to determine the effect of carrier and target position, periodicity and temporal cueing on the population, rather than estimating d’ on each condition separately and feeding the estimated values to a second level analysis. This statistical framework extends multiple linear regression to non-normal data such as count data and binary outcomes and it is more suited to handle extreme cases (100% hits or 0% false alarms). Within this framework, d’ can be estimated by linearly modeling the behavioral outcomes (i.e. ‘yes’ or ‘no’) with a predictor *X* coding for the presence or absence of the target (see right side of Eq (1); and “Target” predictor in Table 2). To fit an equal-variance Gaussian signal detection model an inverse Gaussian (probit) link function is used, where *g* is the link function and *X* represents the presence or absence of the signal.

$$g(E[\text{Pr}\left(Resp{=}^{'}Yes^{'}\right)])=\beta_{0}+\beta_{1}X$$ (1)

**Table 2:** Wilkinson notation of final model in each experiment.

**Exp 1**	d’ ~ Criterion + Target*Rhythm*Carrier*Experiment + (1|Subject) logRT ~ 1+ Rhythm*Carrier*Experiment + (1|Subject)
**Exp 2**	d’ ~ Criterion+ Target*temporalCondition + (1|Subject) logRT ~ 1+ temporalCondition

Criterion is an additional predictor reflecting the intercept (normally notated as 1, here re-parameterized to -1 to reduce correlation between fixed effects (see p 262, [41])

When the signal is absent (i.e. *X* = 0), *β*_0_ provides an estimate of the normal quantile of the false alarm rate. When the signal is present (i.e. *X* = 1), *β*_1_ reflects the difference between hit and false alarm rate on the probit scale (hence, the difference between z-scaled hit and false alarm rates), or d’. The different experimental conditions are then added as predictors, and the estimated d’ for each of these conditions (hence our effect of interest) is described by the interaction term between *X* (‘target’) and the respective condition predictor (see [41]chapter 3.3.5). Unstandardized effect sizes (betas) are reported in units of the dependent variable (d ‘ or logRT), allowing for a meaningful comparison, in line with general recommendations on how to report effect sizes in psychological research [42].

**Fig 2:**
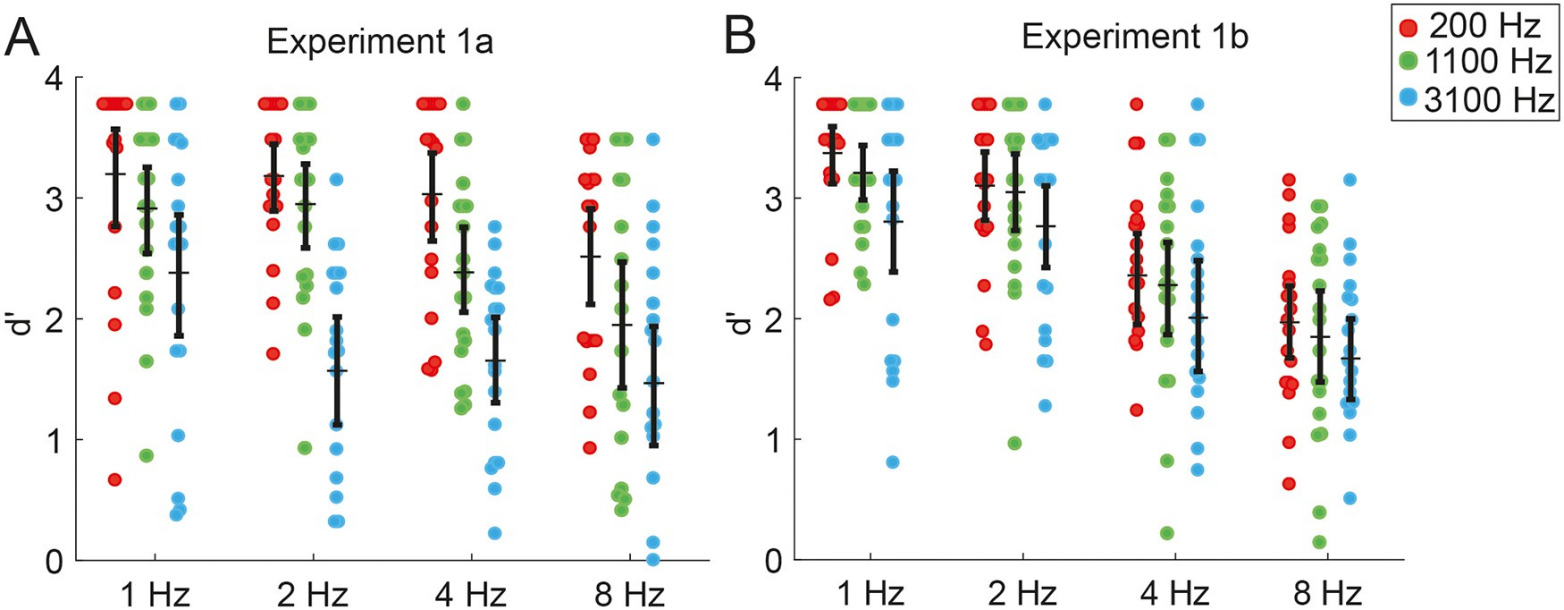
Both, the rhythm and carrier frequency parametrically affect sensitivity. Average d’ of each participant (colored dots). Horizontal line indicates group mean, and errorbars depict bootstrapped 95% confidence intervals (at subject-level). **A** Experiment 1a; target occurred after a constant number of quintets per rhythm. **B** Experiment 1b; target occurred after constant time across rhythms.

For our visualization, we estimated d’ as it is traditionally computed. Standard errors of d’ group effects displayed in Figs 2 and 4 were obtained by non-parametric bootstrap sampling of estimated d’ values, carried out at the subject level (N = 1000). The mean was used as a measure of central tendency around which 95% confidence intervals were created. All planned contrasts were corrected for multiple comparisons, using Bonferroni correction.

## Results

### Comparing periodic sequences at different carrier frequencies (experiment 1)

We investigated the benefits in perceptual sensitivity and reaction times associated with rhythmic sound presentation as a function of the rhythm and the carrier frequency in two experiment variants in which we varied the target position within a sequence (Experiment 1a and 1b). The data from both experiments were fitted using a single large GLMM (see Table 2 for final notation of model).

#### Slow entraining rhythms improve target detection

**Fig 3:**
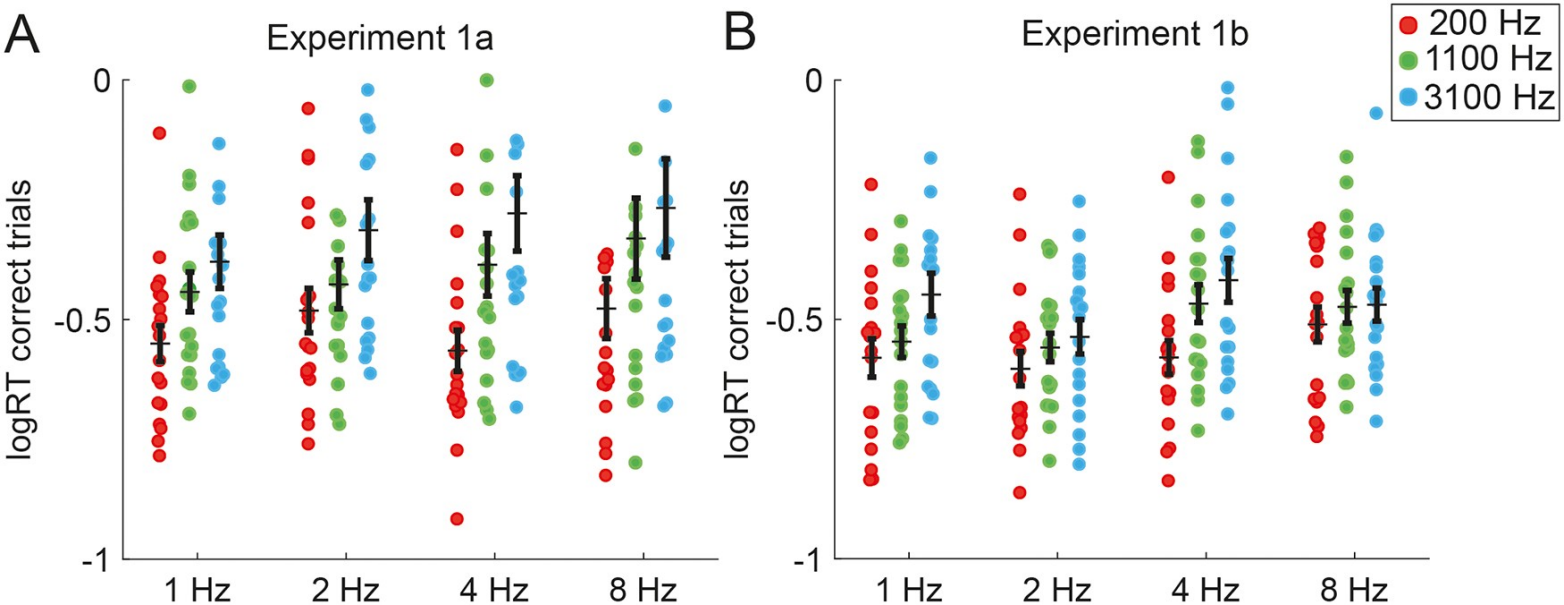
Carrier frequency, but not rhythm, affects reaction times in both experiments. Mean logRT of each participant. Horizontal line indicates group mean, and errorbars depict SEM. **A** Experiment 1a; target occurred after a constant number of quintets per rhythm. **B** Experiment 1b; target occurred after constant time across rhythms.

The analysis showed a significant interaction of experiment and rhythm on d’ (F (3,864) = 4.528, p < 0.01), (see Table 3). Therefore, we analyzed the effect of rhythm separately for experiment 1a and 1b, revealing an effect of rhythm on sensitivity in both, experiment 1a (Fig 2A), F (3,432) = 9.3704.19, p < 0.001) as well as experiment 1b (Fig 2B), (F (3,432) = 26.083, p < 0.001). Follow-up tests showed, for both experiment variants, a parametric effect of rhythm on sensitivity (see Table 4). Counter to our hypothesis of an inverted U-shape where 4 Hz would perform best, we observed a parametric effect of rhythm. The slowest rhythms (1 Hz & 2 Hz) led to significantly higher sensitivity compared to the fastest (8 Hz) in both experiments. In addition, in experiment 1b the slowest rhythms (1 Hz & 2 Hz) led to significantly better sensitivity than 4 Hz as well. (see Table 4 for specific contrasts). We did not observe this parametric effect of rhythm on reaction times (Fig 3) (F (3,431) = 2.498 p >0.05).

**Table 3:** Planned contrasts sensitivity.

	β	F	p
(2 Hz-1 Hz):Experiment	-0.8656	F(1, 864) = 4.66	p>0.05
**(4 Hz-1 Hz):Experiment**	**-1.3884**	**F(1, 864) = 12.82**	**p<0.01**
**(8 Hz-1Hz):Experiment**	**-1.1338**	**F(1, 864) = 8.67**	**p <0.05**
(4 Hz-2 Hz):Experiment	-0.5228	F(1, 864) = 2.63	p>0.05
(8 Hz-2 Hz):Experiment	-0.2682	F(1, 864) = 0.71	p>0.05
(8 Hz-4 Hz):Experiment	0.2546	F(1, 864) = 0.71	p>0.05
200 Hz- 1100 Hz	0.0983	F(1, 864) = 0.18	p>0.05
**200 Hz-3100 Hz**	**0.9364**	**F(1, 864) = 19.42**	**p <0.001**
**1100 Hz-3100 Hz**	**-0.8381**	**F(1, 864) = 14.92**	**p <0.001**

Estimates are in d’. 1 Hz and 1100 Hz are reference categories for dummy coding scheme. Bold values indicate statistically significant results. p < 0.05, Bonferroni corrected. Each row refers to a contrast that interacts with the target predictor.

**Table 4:** Planned contrasts of rhythm per experiment.

	contrasts	β	F	p
**Experiment 1a**	(2 Hz-1 Hz)	0.0750	F(1, 432) = 0.10	p>0.05
	(4 Hz-1 Hz)	-0.4874	F(1, 432) = 4.13	p>0.05
	**(8 Hz-1 Hz)**	**-1.0152**	**F(1, 432) = 19.29**	**p <0.001**
	(4 Hz-2 Hz)	0.5624	F(1, 432) = 5.24	p>0.05
	**(8 Hz-2 Hz)**	**1.0903**	**F(1, 432) = 21.13**	**p<0.001**
	(8 Hz-4 Hz)	0.5279	F(1, 432) = 5.07	p>0.05
**Experiment 1b**	(2 Hz-1 Hz)	-0.7902	F(1, 432) = 6.11	p>0.05
	**(4 Hz-1 Hz)**	**-1.8744**	**F(1, 432) = 37.83**	**p<0.001**
	**(8 Hz-1 Hz)**	**-2.1465**	**F(1, 432) = 48.59**	**p<0.001**
	**(4 Hz-2 Hz)**	**1.0842**	**F(1, 432) = 26.97**	**p<0.001**
	**(8 Hz-2 Hz)**	**1.3563**	**F(1, 432) = 40.41**	**p<0.001**
	(8 Hz-4 Hz)	0.2721	F(1, 432) = 2.05	p>0.05

Estimates are in d’. 1 Hz and 1100 Hz are reference categories for dummy coding scheme. Bold values indicate statistically significant results. p < 0.05, Bonferroni corrected. Each row refers to a contrast that interacts with the target predictor.

#### Carrier frequency of the stimulus affects target detection and reaction time

We observed a parametric main effect of carrier frequency on sensitivity F (2,864) = 12.2, p<0.001). Participants were more sensitive in detecting a target when listening to a sequence with a 200 Hz carrier compared to the high carrier frequency at 3100 Hz (beta = -0.9364; F (1, 864) = 19.42, p = <0.001), but also when comparing the 1100 Hz carrier against 3100 Hz carrier (beta = -0.8381; F (1, 864) = 14.92, p<0.001). The difference in sensitivity between the 200 Hz and 1100 Hz was not significant (beta = 0.0983; F (1, 864) = 0.18). In addition, we observe that the carrier frequency had a significant effect on logRT (F (2,431) = 7.539, p< 0.001). Comparisons showed that the lowest carrier frequency led to significantly faster responses compared to the middle carrier (beta = -0.1078; F (1, 431) = 5.87, p<0.05), and the highest carrier frequency (beta = -0.1709; F (1, 431) = 14.74, p<0.001) (Fig 3).

#### Carrier Frequency and rhythm do not interact in their effects on sensitivity and reaction time

The interaction of rhythm by carrier was not significant in reaction times (F (6,431) = 1.125, p > 0.05) or sensitivity (F (6,864) = 2.0454, p > 0.05).

### Effect of (a)periodic sequences and a temporal cue at perceptual threshold (experiment 2)

Experiment 2 compared the effect of periodic predictable and aperiodic unpredictable sequences of 6 second length to a temporal cueing condition with a cueing interval matching the ISI of the sequences (500ms). We fitted two GLMMs for the reaction time data and d’ data respectively. Each model consisted of the fixed-effect within-subject factor *temporal structure* (3 levels; periodic, aperiodic, cue).

#### Temporally predictable stimulation (through a periodic rhythm or a cue) improves sensitivity

**Fig 4:**
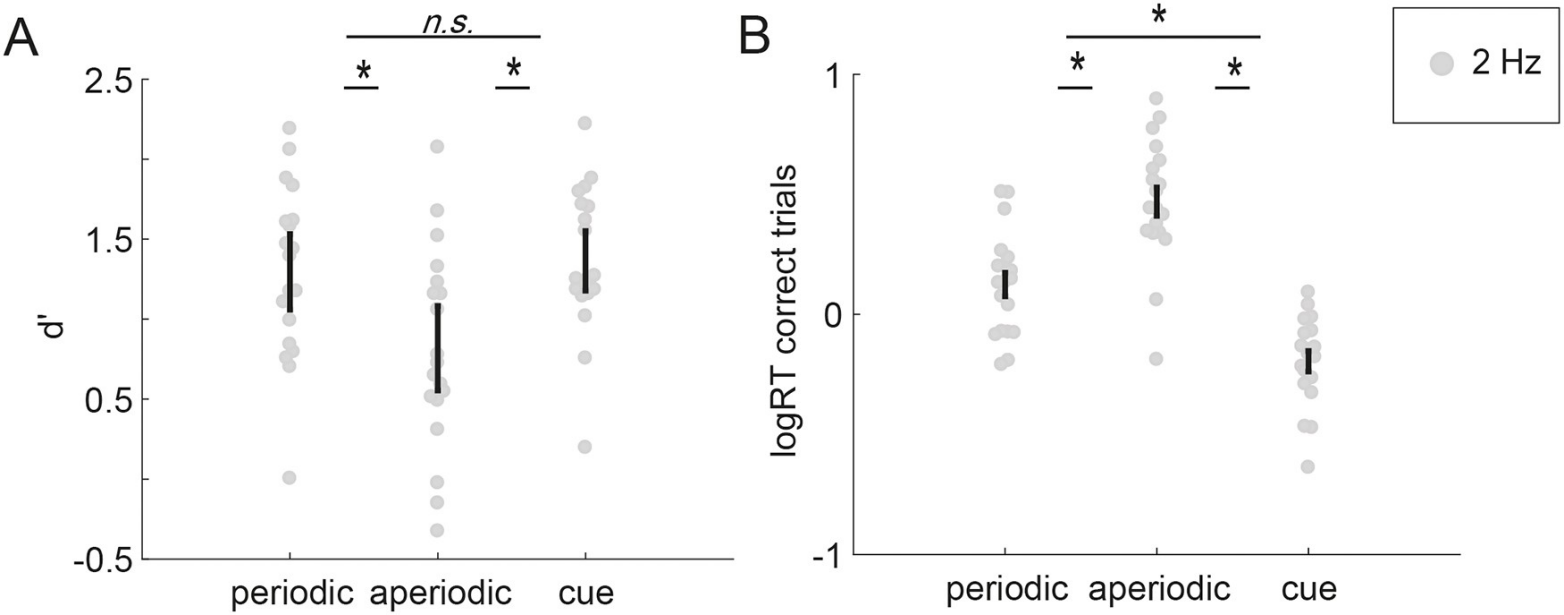
Experiment 2. When controlling for task difficulty, periodicity and a temporal cue improve hit reaction time and d’ compared to the aperiodic condition. TS size between 1.5-7ms (70% detection threshold). See S3 Fig for average TS size. **A** d’ per participant. Errorbars depict bootstrapped confidence intervals (at subject-level). **B** Mean logRT of each participant. Errorbars depict SEM.

We compared target detection sensitivity in three temporal context conditions: predictable periodic, unpredictable aperiodic, (predictable) temporal cue. We found d’ to vary significantly as a function of temporal context (Fig 4A, F (2, 114) = 52.663, p< 0.001). Comparisons between the conditions revealed significant differences in d’ between the predictable periodic and unpredictable aperiodic sequences (beta = 0.5903; t (1, 114) = 7.95, p< 0.001), as well as between the aperiodic sequence and the temporal cue (beta = 0.7154; t (1, 114) = 9.3612, p<0.001). The difference in d' between predictable periodic sequences and the temporal cue was not significant (beta = 0.1251; F (1, 114) = 2.49, p>0.05).

#### Predictable temporal cue and a predictable periodic sequences improve reaction time compared to an aperiodic unpredictable sequence

The analysis of reaction times yielded a significant, yet different pattern between conditions (Fig 4B, F (2, 57) = 48.1, p< 0.01). Comparisons revealed faster correct responses for periodic predictable than aperiodic unpredictable sequences (beta = -0.3317; t (1, 57) = -4.894, p< 0.001). Moreover, participants responded faster to temporally cued targets than periodic rhythms, (beta = -0.3331; F (1, 57) = 24.15, p<0.001) as well as aperiodic (beta = -0.6647; t (1,57) = 9.808, p<0.001) rhythms. Thus, temporal predictability (whether through a cue or a periodic rhythm) led to an improvement of auditory sensitivity, while participants additionally benefit in their response times from the periodicity of stimulation compared to aperiodic stimulation, and a temporally predictable cue leading to the fastest response.

## Discussion

Using a temporal shift detection task we asked how the rate and the carrier frequency of a predictable periodic rhythm influence both reaction time and sensitivity of perceptual decisions. Moreover, we asked whether the predictive advantage derived from periodic stimulation is larger than that afforded by aperiodic stimulation or a single temporal cue. The data show that (1) the largest sensitivity improvement is observed for the slowest rhythm (1 Hz); (2) sensitivity improvement is larger for low-frequency (200 Hz) carriers compared to 1100 Hz and 3100 Hz carriers; (3) periodic stimulation significantly reduces reaction times compared to aperiodic stimulation (speeded responses were observed both at perceptual threshold as well as above threshold during the pilot experiment); (4) a response to a temporal cue is faster than a response to periodic stimulation (5) periodic stimulation and cueing significantly increase sensitivity compared to aperiodic stimulation.

### Experiment 1. Largest sensitivity improvements occur at slowest rhythm

Psychophysical findings show that sensitivity towards amplitude modulation detection of noise is highest for humans in the (speech) range of 2–4 Hz, while highest for macaques in the range of 30–60 Hz [43]. These and other findings have led to the notion that the human auditory cortex is considered to be speech-ready, therefore, we expected a peak in perceptual sensitivity with periodic sound presentation around 4 Hz. However, our results show that listeners’ performance was highest at a slow rhythm and decreased with increasing rate of rhythm. A similar pattern of preference for slow rhythms (e.g. 2 Hz) as opposed to faster rhythms (8 Hz) has also been shown for cortical synchronization (entrainment) to speech in noise. Phase-locking of neural activity to speech embedded in noise decreased from low (2 Hz) to high (8 Hz) frequencies, correlating with speech intelligibility [44]. This pattern resembles the linear decrease across rhythms observed here and supports the predominant role of delta band frequencies in auditory processing.

Moreover, we speculate that our findings may relate to the nature of the task and stimuli we used. The acoustic features of the isochronous stimuli may be closer to music and its temporal modulations than to the modulations inherent in speech. Temporal modulations in western music peak between 0.5 and 3 Hz, depending on the instrument and may contribute to a preference for slower modulations. As to why the peak of the modulation spectrum in music may be lower than that of spoken speech, it has been suggested that music like language is limited by the dynamic rate of movement of the effector (i.e. the frequency range where movement is most efficient; usually hands and arms in the case of music and articulators in the case of language) [21]. Slow rhythms (1 Hz and 2 Hz) approximate the rate of spontaneous, hence most efficient, motor tempo (around 1.5 Hz for adults) as measured by spontaneous tapping-tasks [45]. A recent review substantiates the link between auditory processing and the motor system, suggesting a downward propagation of temporal predictions from the motor system involving delta-oscillations that shape auditory perception by imposing temporal constraints [46].

Lastly, we show that reaction times were not modulated by different rhythms. Preparatory response processes are typically studied in foreperiod (FP)—reaction time experiments, in which it is a classical finding that both the duration of the FP (usually in the range of seconds) as well as the variability of FP across trials within a block have a considerable effect on reaction times [33]. In the present study, ISI within a block were constant (i.e. low variability across trials), allowing the participant to prepare a motor response equally probable across conditions. Moreover, the absence of a difference in reaction times suggests that the time-range tested here allowed for non-specific (motor) preparations across all rates. Despite no difference in reaction times across rates, a difference in sensitivity across rates was observed, highlighting the perceptual benefit of rhythmic sound presentation, especially for slower rhythms.

### Experiment 1. Carrier-dependent improvement of sensitivity and reaction times

Surprisingly, we found that the sensitivity decreased with increasing the carrier frequency. Based on the literature on temporal modulation processing in humans, we would expect higher sensitivity for higher carrier frequencies, as sounds are encoded by auditory spectral filters (tonotopic mechanism). These spectral filters are narrower at lower frequencies and wider at higher frequencies and limit the temporal resolution of the auditory system. Therefore detection performance of a temporal shift should decrease at lower carrier frequencies where the bandwidth is narrower [47]. Indeed, increasing modulation detection thresholds for decreasing center frequencies have been observed [48,49]. We controlled for this effect, by adjusting the stimuli to have equivalent rectangular bandwidth (ERB) and equivalent perceived intensity. Despite this equalization we observe an effect of carrier frequency. We suggest that the perceptual benefit at the low carrier frequency may be a product of temporal coding mechanisms. Phase locking up to 250 Hz has been observed in human intracortical recordings using click trains [50]. This temporal encoding seems to provide a perceptual benefit when making judgments about the presence of a temporally shifted target thereby improving sensitivity. Additionally, this benefit in sensitivity for the lowest carrier frequency was accompanied by an increase in response speed. Simpson, Reiss and McAlpine [25] estimated sensitivity to a range of amplitude modulation frequencies (0.5 Hz to 33 Hz) across a large number of frequency carriers including and beyond the range of carriers tested here. Their results suggest that at low carrier frequencies cortical modulation filters are most sensitive to slow modulation rates, similar to the rates used here (1–8 Hz). They speculate that such a frequency-dependent modulation tuning is related to the neural processing of acoustic properties of speech [51]. Lastly, these findings suggest that behavioral effects of entrainment, may depend on the type of spectral stimulation used for entraining and probing, which may be especially relevant in the context of spectral tasks [26].

### Experiment 1. Effect of rhythm not confounded by target position

The number of preceding quintets was varied between experiment 1a and 1b. In experiment 1a we kept the number of preceding quintets until the TS constant across rhythms. This systematically reduced the time until a target appeared with increasing rhythm. In experiment 1b, we kept the time at which targets appeared constant across rhythms, thereby presenting an increasing number of preceding quintets with increasing rhythms prior to presenting a target quintet. Our rationale to assess and compare these two experiment versions being, that the strength of entrainment might increase with additional repetitions. The effect of rhythm would then be confounded by the systematic effect of target position in a sequence. Indeed, we show in experiment 1a as well as 1b an effect of rhythm. By having controlled the position of the target across the different presentation rates we therefore conclude that there is a difference between rhythms and said effect was not confounded by the systematic effect of target position in a sequence. In experiment 2 targets embedded in aperiodic and periodic sequences occurred late within a sequence (similar to Experiment 1b). It would be interesting to see what the effect of aperiodic and periodic sequences is when targets are presented earlier. We may speculate that this would further enhance the detection difficulty of the task enhancing the benefit of periodicity.

### Experiment 2. Effects of periodicity and cueing diverge in logRT and sensitivity

The results of experiment 2 show that both perceptual sensitivity and reaction times are improved when stimuli are presented in periodic rhythms compared to aperiodic rhythms, when using a temporal detection task at perceptual threshold. Note that these effects are only apparent when controlling for task difficulty. In a pilot study, where this was not done, we did not observe an effect of periodicity on sensitivity (see S1 File). The results of experiment 2 support earlier findings reporting a benefit of predictability in periodic over aperiodic stimulation [4–7,52–55]. This is in line with the idea of oscillatory entrainment and dynamic attending theory [56] and highlights the relevance of using a task with sufficient difficulty, in contrast to the pilot. However, this finding points to a more general question of the benefit of entrainment in everyday life as most stimuli we encounter are seldom at perceptual threshold.

In addition, we were interested in contrasting the benefit in reaction times and sensitivity of a predictable periodic rhythm to a predictable (but not periodic) temporal cue. We expected a benefit in reaction times similar to [6] (albeit different) showing a benefit of a periodic predictable sequence over an aperiodic, predictable condition of increasing tempo. Moreover, we expected a benefit in sensitivity for the periodic rhythm compared to the temporal cue. We show in experiment 2, that a temporal cue enables a participant to respond significantly faster than a periodic rhythm, while there is no difference in perceptual sensitivity between these two forms of temporal structure. The cue predicts target occurrence with a 75% validity (25% of trials were catch-trials without a target). Similarly, the periodic rhythm allowed participants to predict when a subsequent target may occur with a similar validity for the trial. Yet participants were faced with the additional uncertainty as to which quintet within a sequence may contain the target. This uncertainty may be the reason for the observed slowing of reaction times in the periodic condition compared to the cue. The greater effectiveness of the temporal cue compared to the rhythm in terms of reaction time suggests that the temporal cue induced a more confident temporal expectation, in line with the finding that target-occurrence uncertainty impairs reorienting, thereby lengthening of reaction times [57]. It would be of interest to compare these two conditions under similar uncertainty of target occurrence.

Interestingly though, despite said larger uncertainty the periodic condition is not significantly worse than the temporal cue in terms of sensitivity, therefore we suggest that a benefit of a periodic rhythm to some extent countered the increased uncertainty. Ten Oever et al. have shown that entrainment of low-frequency oscillations in the delta—range serves a mechanistic role in enhancing perceptual sensitivity of subthreshold periodic, predictable sound sequences compared to aperiodic sequences [18]. Under the hypothesis that oscillations align more efficiently to a rhythmic structure as compared to a single interval, it is surprising that the periodic sequences here did not result in a sensitivity benefit over the temporal cue. See for instance [58,59]showing an accuracy benefit of periodic sequence over cue in duration estimation. We speculate that the additional uncertainty of when within a sequence a target may occur, may have countered a benefit of the entraining rhythmicity of the sequence. Again, it would be of interest to compare instances of a predictable periodic rhythm and a predictable temporal cue conveying the same uncertainty. We would then predict sensitivity of the periodic predictable condition to be higher than the temporal cue condition.

At the neurophysiological level, we speculate that such a mechanism might be implemented by a more flexible phase reset model of neuronal oscillations [15,60,61], (see [62], for a recent review), in which the motor system tracks temporal regularities [46]. Further research will be necessary to elucidate the mechanism and nature of top-down predictions and how these affect auditory perception.

## Conclusion

We show that overall temporal modulations in the range of 1–8 Hz are better processed at lower carrier frequencies, as measured by reaction times and sensitivity (experiments 1a and 1b). Additionally, the same results point to the perceptual benefit of slow rhythms (1 and 2 Hz) over faster ones (4 and 8 Hz). The regularity of rhythms enables the use of prediction to make more precise inferences about when we should expect to find a target embedded within the stream and, as a result, improve detection performance. Indeed, we show in experiment 2 a perceptual benefit of periodic predictable sequences over aperiodic unpredictable sequences in terms of reaction times and sensitivity (the latter only present when using a sufficiently difficult task). Crucially, in experiment 2 we show that the predictive value of a cue and that of a temporal rhythm do not differ in terms of the sensitivity in detecting a target, albeit it has to be noted that the periodic condition contained a larger uncertainty where the target would appear. These findings encourage us to reflect on what the perceptual benefits of periodicity and predictability respectively are, as these effects may diverge when teased apart using different tasks thereby allowing to make assumptions about the underlying mechanisms involved. Here we showed that both the cue and the rhythm induce confident temporal expectancies about the future occurrence of targets to effectively prepare and allocate attentional resources. Taken together, we may speculate that multiple processes may co-occur that facilitate the processing of rhythmic and predictable stimuli, in which oscillations form an intrinsic temporal constraint, controlled by temporal predictions. Potentially, cueing effects occur due to a single phase-reset of ongoing oscillations and similarly a rhythmic benefit occurs due to either a stimulus driven entrainment of oscillations or repeated top-down phase resets.

## Supporting information

S1 File (DOCX)
