# Supplementary material for: Behavioral effects of rhythm, carrier frequency and temporal cueing on the perception of sound sequences
Source: PLoS One. 2020 Jun 5;15(6):e0234251. doi: 10.1371/journal.pone.0234251 (PMC7274376; doi:10.1371/journal.pone.0234251)
Supplement: S1 File — (DOCX) [file pone.0234251.s001.docx]

Pilot Experiment 2

**Materials & Methods**

*Participants*

20 subjects (11 females, 9 males) participated in the pilot experiment. The Ethics Review Committee of the Faculty of Psychology and Neuroscience (ERCPN) at Maastricht University granted approval for all studies and all participants gave informed consent.


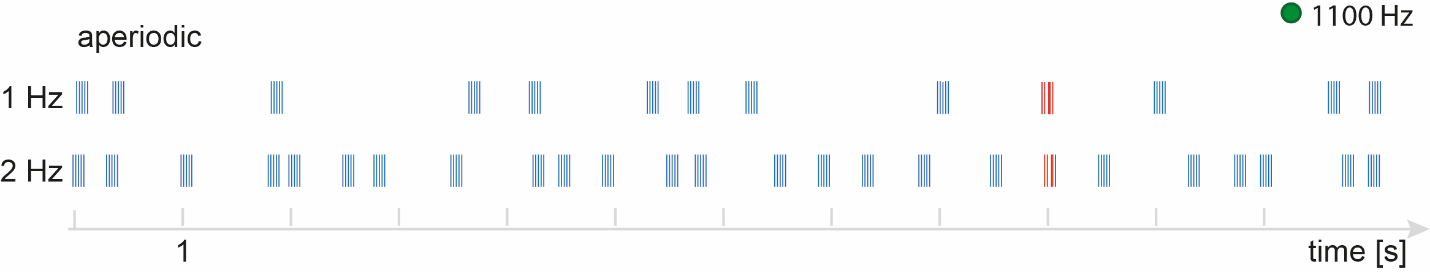
 **Figure S1. Stimuli pilot.** Stimuli were presented with a carrier frequency of 1100 Hz. Two aperiodic conditions were presented with sequences of 12s length that had a similar number of quintets as periodic sequences at 1 Hz and 2 Hz of experiment 1.

*Stimuli & Design*

Similar to experiment 1, participants were asked to detect targets embedded in sound sequences of 12s length. The participants performed 80 trials of the target detection task on aperiodic stimuli. See methods experiment 2 of the main text on details of aperiodic stimuli. Stimuli were presented in blocks of 16 trials, in which the average rhythm was constant. The size of the temporal shift (TS) was fixed at 6ms occurring after a fixed time (similar to experiment 1b). During a trial and up to 1 second after a quintet sequence finished, participants could press a button upon detecting a TS or another button at the end of a sequence indicating they did not perceive a TS.

*Statistical analysis*

For details on statistical analysis see main text.

**Table S1. Wilkinson notation of final model in pilot experiment**

| Pilot | d’ ~ Criterion + Target*Rhythm*Experiment + (1 + Target:Rhythm\|Subject)  logRT ~ 1 + Rhythm*Experiment + (1 \|Subject) |
| --- | --- |

Criterion is an additional predictor reflecting the intercept (normally notated as 1, here re-parameterized to -1 to reduce correlation between fixed effects (see p 262, Knoblauch & Maloney, 2012))

**Results**

We examined the effect of (average) rhythm on target detection, by comparing periodic predictable sequences (matching conditions from experiment 1b) to aperiodic unpredictable sequences of quintets presented in the pilot.

***Comparing aperiodic (pilot) and periodic sequences (experiment 1b) at 1 and 2 Hz above perceptual threshold***

Two GLMMs with *rhythm* as within-subject factor (2 levels; 1 Hz, 2 Hz) and *experiment* as between-subjects factor were created to analyze log-reaction times and sensitivity data, comparing aperiodic and periodic presentation (Fig S1).

**
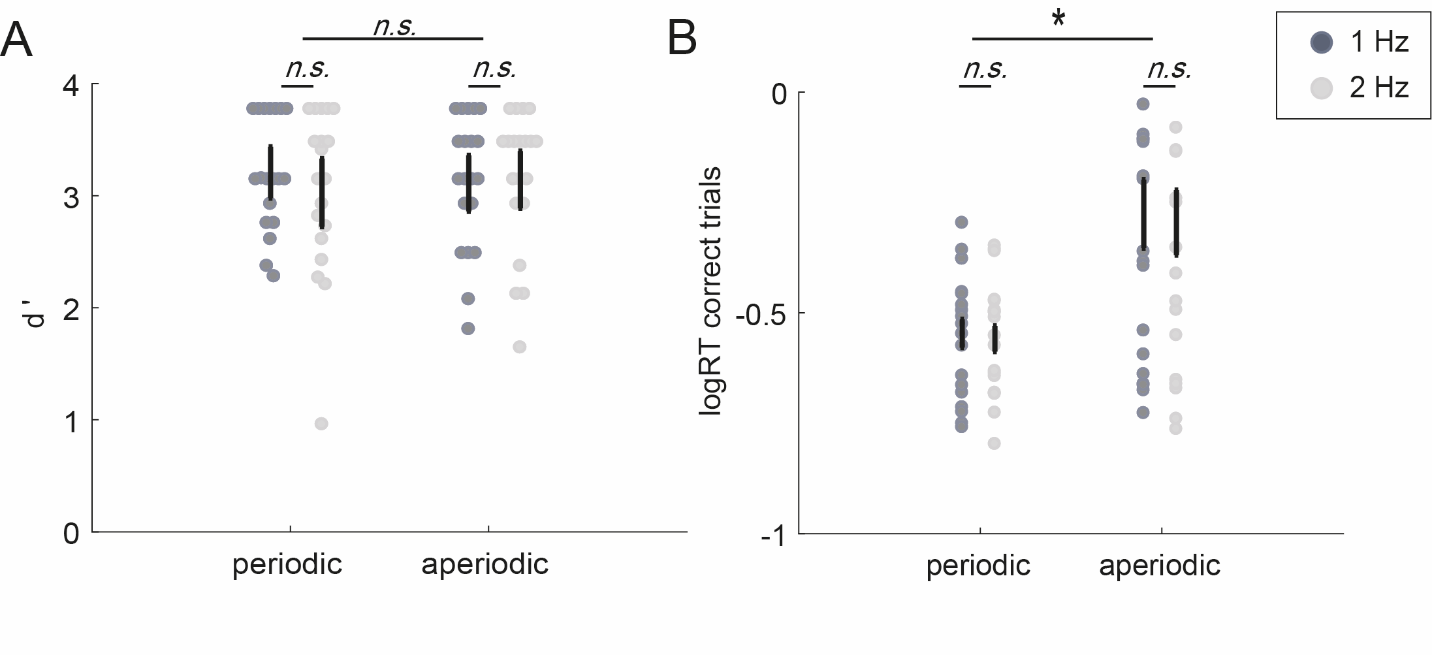
**

**Figure S2. Periodicity improves hit reaction time but not d’ when TS fixed at 6 ms.** Comparing matching conditions from experiment 1b (periodic) to experiment 2 (aperiodic). No difference between rhythms (1 & 2 Hz). **A** d’ per participant. Errorbars depict bootstrapped confidence intervals (at subject-level). **B** Mean logRT of each participant. Errorbars depict SEM.

*Periodicity improves reaction times but not sensitivity*

Periodic stimulation improved reaction times when compared to the aperiodic stimulation (Fig S1 A, beta = 0.2708; (t (1, 74) = 3.336, p < 0.01). We did not find an effect of periodicity on perceptual sensitivity (Fig S1 B, beta = -0.0211; (t (1,148) = -0.051 p>0.05), when a detection task using a fixed target of 6ms was used (Fig 4; Experiments 1 and 2). We did not observe significant differences between the 1 and 2 Hz rhythm in either experiment in their effect on reaction times (beta = -0.012; t (1, 74) =-0.509, p>0.05) or on d’ (beta= -0.4886; t (1, 148) =-1.160 p>0.05).

**Discussion**

The pilot results are discussed in the main body of the paper.


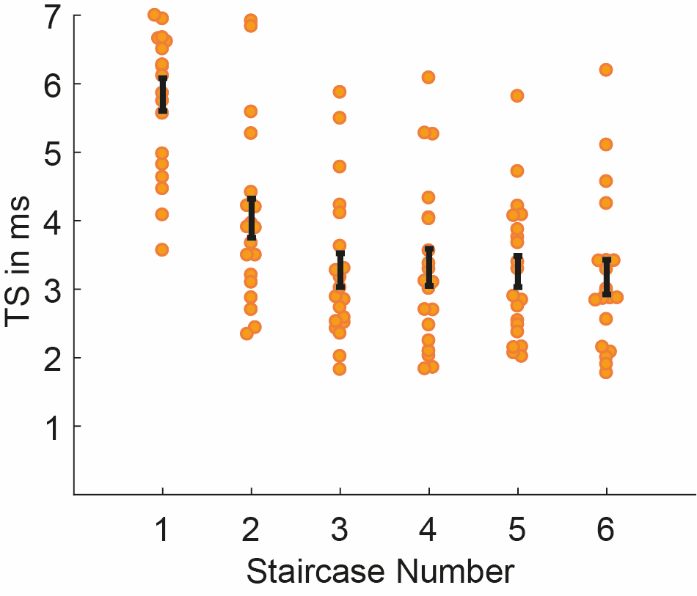


**Figure S3. Decrease of TS size over course of experiment 2.** TS size per participant of in total six staircase blocks. Each staircase preceded a trial block of experiment 2, ensuring sufficient task difficulty in the latter. Group average of to be detected TS decreases over the course of the experiment. During the staircase the TS varied on a fixed step-size of 10 logarithmically spaced steps between 7ms and 1.5 ms. The termination criterion was after 200 trials or 15 reversals. Errorbars depict SEM centered on group mean.
